# Prevalence of occupational respiratory disease and its determinants among workers in major industrial sectors in Malaysia in 2023

**DOI:** 10.1038/s41598-025-10365-8

**Published:** 2025-07-16

**Authors:** Muhamad Syazni Asraff, Meram Azzani, Mohd Ridzuan Anuar, Ahmad Faiz Azhari Noor, Ahmad Fitri Abdullah Hair, Ayu Suriawaty Bahkia, Mohd Supian Hassan, Siti Munira Yasin

**Affiliations:** 1 Public Health Medicine Department, Faculty of Medicine, Universiti Teknologi Mara (UiTM), Sungai Buloh, 47000 Selangor Malaysia; 2 Occupational Health Division, Department of Occupational Safety and Health, Ministry of Human Resource, Putrajaya, Malaysia; 3 Indah Water Konsortium Ltd, Kuala Lumpur, 50490 Malaysia; 4 Pharmaniaga Logistics Sdn Bhd, Shah Alam, 40000 Selangor Malaysia

**Keywords:** Occupational respiratory disease, Major industrial sectors, Prevalence, Determinants, Malaysia, Diseases, Risk factors

## Abstract

In Malaysia, rapid industrial growth has increased workers’ exposure to occupational respiratory hazards which consequently leads to an increase in the prevalence of occupational respiratory disease (ORD) among workers. Therefore, this study aims to fill this gap by investigating the prevalence of ORD and its determinants among workers. This is a cross-sectional study utilizing secondary data from the National Occupational Disease and Prevention 2023 (NODIP) database involving a total of 111,028 participants. Suspected ORD was defined by the British Medical Research Council (BMRC) questionnaire criteria. Both descriptive and inferential statistics were utilized in this study. Multivariable logistic regression analysis was used to find the determinants of ORD. The overall prevalence of ORD was found to be 8.7%. Most of studied factors were significant determinants of ORD. However, the factors of highest odds ratio (OR) were working in the mining and quarrying (AOR: 14.81; 95% CI: 11.74–18.68; *p* < 0.001), being of Malaysian nationality (AOR: 4.48; 95% CI: 3.78–5.32; *p* < 0.001) and working more than 12 hours per day (AOR: 2.18; 95% CI: 1.94–2.45, *p* < 0.001). The findings of this study highlight the importance of regular assessment of the risk of exposure to noxious agents in the workplace and to develop measures for preventing potential adverse effects.

**Supplementary Information:**

The online version contains supplementary material available at 10.1038/s41598-025-10365-8.

## Introduction

Globally, occupational respiratory diseases (ORD) are significant public health concern. The World Health Organization (WHO) estimates that 1.9 million people die annually from job-related respiratory illnesses which also severely impact quality of life, making ORD a leading cause of work-related sickness and mortality worldwide^1^. Due to exposure to a variety of chemicals, including metal particles, endotoxins, pesticides, and different air pollutants, workers in many industrial sectors are more likely to experience respiratory issues^1^.

According to Murgia, Akgun^2^ occupational lung illnesses are specifically characterised as conditions that are either induced, exacerbated, or intensified by exposure to certain substances or conditions on the job. These exposures may involve inhalable substances such as coal mine dust and silica, as well as fumes, vapours, gases, chemicals, metals, and infectious pathogens^2^ leading to a serious negative effect on workers’ quality of life and productivity.

A study by Jumat, Hayati^3^ reporting that there is a worrying increasing trend of ORD in Malaysia. Numerous studies have established that socio-demographic, occupational, and organizational factors significantly contribute to the development of ORD among industrial workers^3^. However previous studies did not investigate all the seven major industrial sectors simultaneously, they were of smaller sample size and not nationally representative, limiting the comprehensiveness of their findings^4–7^. This study however, addressing the gap by researching ORD prevalence throughout all major sectors in one study, providing an extensive national overview and highlighting the novelty of a sector-wide analysis.

Socio-demographic factors such as age, gender, race, level of education, medical illness, and smoking habits influence susceptibility to respiratory conditions. For instance, smoking workers and those with medical illnesses are more prone to developing respiratory issues due to prolonged exposure to harmful substances^8^.

Occupational factors, such as the type of industry, duration of working hours per day, duration of employment, and specific job roles, significantly influence the level of exposure to respiratory hazards in the workplace. Research has emphasized the importance of identifying and attributing occupational exposures as causes of respiratory disease development, progression, and exacerbation to facilitate accurate diagnosis and management of ORD^9^.

Organizational factors such as the dissemination of occupational safety and health (OSH) knowledge, training effectiveness, and adherence to personal protective equipment (PPE) protocols significantly influence ORD. The correct utilization of PPE and strict compliance with its usage are pivotal in mitigating respiratory risks in the workplace. Recent research, exemplified by Azizi, Varasteh and Esmaeili^10^, underscores the critical role of consistent PPE compliance and other safety protocols in minimizing workplace hazards^10^.

Despite acknowledging the global burden of ORDs, Malaysia’s inadequate surveillance and epidemiological data hinder the establishment of targeted initiatives and worker protection policies. Awaluddin, Mahjom^11^ claim that a lack of awareness among employers and employees, as well as insufficient reporting procedures, are the main reasons why many occurrences of ORD are unreported. Furthermore, workers in Malaysia’s broad industrial landscape which includes everything from manufacturing to agriculture are exposed to a range of respiratory dangers; yet, the particular risks connected to these industries are not well-documented^12^. Our research intends to close this gap by (1) determine the prevalence of ORD across all seven major industrial sectors in Malaysia, (2) identify socio-demographic, occupational, and organizational determinants of ORD using a national dataset (NODIP 2023).

## Methodology

This is a cross-sectional study utilizing secondary data from the National Occupational Disease Prevention Programme (NODIP) 2023. We analysed responses from 114, 932 participants throughout the sectors of industries in Malaysia. NODIP was led by the Department of Occupational Safety & Health (DOSH) in collaboration with the Occupational Health Department, UiTM Campus Sungai Buloh, to detect occupational diseases early in workplaces in Malaysia. It is a three-year project that includes a survey of workers from all 13 states in Malaysia. Data was collected using a multistage stratified sampling method from 500 companies including seven critical industrial sectors through a self-reported questionnaire using Google form. The database contains 466 variables. Independent variables included socio-demographic, occupational, and organizational factors, and symptoms of ORD, while the dependent variable was occupational respiratory disease.

### Study population

The study population comprises all workers employed in the following major industrial sectors in Malaysia: manufacturing, construction, mining and quarry, trade wholesale retail, hotels and restaurants, agriculture, fishery, forestry, and utilities (gas, electrical, water, and sanitation services).

### Sampling methods

Participants for this study were selected through universal sampling from the secondary NODIP database in 2023.

### Inclusion criteria and exclusion criteria

Inclusion criteria encompass adult individuals aged 18–50 years, with employment durations exceeding 1 year, and affiliated with companies registered by the Registration of Company (ROC). Exclusion criteria involve workers with established diagnoses of chest trauma, prior pulmonary surgery, and those diagnosed with lung diseases leading to permanent reduction in lung function.

### Study instruments and tools

The study utilizes secondary data from the NODIP 2023 database, comprising four sections: socio-demographic, occupational factors, organizational factors, and ORD. The socio-demographic questionnaire includes age, sex, marital status, nationality, race, level of education, smoking status, vape user, medical illness, allergic history, and asthma history. Occupational factors include, working hours per day, closed space, confined space, crowded space, and seven different major industrial sectors (manufacturing, construction, mining, and quarry, wholesale and retail, agriculture, fishery, and forestry, utilities, and hotel and restaurant). Organizational factors include knowledge of OSH, knowledge of PPE, training on PPE, and usage of PPE. The outcome of the study on ORD was assessed using the British Medical Research Council Questionnaire (BMRCQ). The BMRCQ is a well-validated tool known for its effectiveness in detecting respiratory conditions in various populations.

### Validation and reliability

The BMRCQ has undergone extensive validation and is recognized for its high sensitivity and specificity. The sensitivity of the questionnaire ranges from 65 to 91%, while its specificity ranges from 85 to 96%. These metrics indicate that the BMRCQ is both a reliable and accurate instrument for the assessment of respiratory diseases^13^. The questionnaire is structured into four domains, each containing four questions, totalling 16 questions, focusing on cough, phlegm, chest tightness, and chest pain. Each question within these domains is tailored to gather specific information about the respective symptom (onset and timing, frequency and duration, severity and progression and work-relatedness) with yes and no answer. Together, these questions form four essential components that aim to characterize the symptoms comprehensively. Onset and timing questions are asked to determine if the symptom starts only in the morning or continues throughout the day. Frequency and duration questions assessed whether the symptom occurs on most days and persists for at least three months of the year. Severity and progression question investigates if the symptom is worsening and lasts for three weeks or more each year. Work-relatedness questions check if the symptom alleviates once the individual is away from their work environment. According to “Current Diagnosis & Treatment: Occupational & Environmental Medicine,” by^14^ at least one of the following criteria must be met to attribute symptoms to work hazards: the symptoms were caused by work hazards, they worsen at work, they improve during off days, or co-workers demonstrate the same symptoms^14^. The presence of one or more symptoms (cough, phlegm, chest tightness, chest pain) and at least one work-related domain in the survey confirms the presence of ORD, you can find the questionnaire in Supplementary File 1.

### Data management

Data extraction involves filtering the BMRCQ questionnaire data from the NODIP database and identifying relevant variables. Data were stored securely and protected using stringent protocols. The present study examined 22 variables, identified through a comprehensive literature review, as significant factors contributing to the development of ORD among industrial workers.

### Data cleaning

Data will be transferred to IBM SPSS Statistics 29.0 software, checked for completeness and missing values, and handled accordingly, including listwise deletion if necessary. From the initial pool of 114,932 respondents, 2603 were excluded due to not meeting the inclusion criteria. This resulted in 112,329 eligible participants. Following data cleaning and analysis for missing information, an additional  1301 participants were excluded. Therefore, the final dataset for analysis comprised 111,028 participants.

### Data analysis

Statistical analyses were performed using the Statistical Package for the Social Sciences (SPSS) version 29. Data were summarised using mean and standard deviation for continuous variables and frequency and percentages for categorical variables. To examine factors associated with ORD, simple logistic regressions were used to estimate the crude odds ratio (COR) with its 95% confidence interval (CI). Factors with p-value < 0.25 identified from univariate analyses were included in the adjusted multivariable logistic regression model to find out the determinants of ORD, and the adjusted odds ratios (AORs) and their 95% CIs were reported. Model fitness was assessed via Hosmer-Lemeshow test (*p* > 0.05), and multicollinearity was checked using Variation Inflation Factor (VIF < 10).

### Ethics approval and consent to participate in this study

Ethical approval was obtained from Universiti Teknologi MARA (UiTM) research ethics board committee and was conducted in accordance with the Declaration of Helsinki principles. Informed consent was obtained from all study participants during the initial NODiP data collection.

## Results

Table 1 shows that 111,028 workers were included in this study, with a mean age of 34 (SD+/- 10 years). Most subjects are male (61.1%) and married (58.5%). The Malaysian workers comprised 89.4% of respondents while non-Malaysians are 10.6%. Races include Malay, 72.3%, Chinese 6.6%, Indian, 5.6%, and others 15.6%. The workers’ level of education was classified as tertiary (50.6%), secondary (43.2%), and primary (6.2%). There were 21.8% smokers, and among those who smoked, 84.1% of them smoked for less than 5 years and 16.3% vaped. In addition, 29.6% of workers have medical illnesses, 14.1% of them are asthmatic and only 4.5% have allergies.

**Table 1:** Socio-demographic factors and health-related factors among study participants (*n* = 111,028).

Independent variables		Mean (SD)	Frequency (%)
Age (years)		34.74 (10.04)	
Gender	Male Female		67,821 (61.1) 43,207 (38.9)
Marital Status	Married Not Married		64,957 (58.5) 46,071 (41.5)
Nationality	Malaysian Non-Malaysian		99,223 (89.4) 11,805 (10.6)
Race	Malay Chinese Indian Others		80,228 (72.3) 7,276 (6.6) 6,169 (5.6) 17,355 (15.6)
Level of Education	Tertiary Secondary Primary		56,141 (50.6) 47,991 (43.2) 6,896 (6.2)
Smoking status	Smoker Non-smoker		24,239 (21.8) 86,789 (78.2)
Vape status	Vape user Non-vape user		18,120 (16.3) 92,908 (83.7)
Comorbidity	Yes No		32,811 (29.6) 78,217 (70.4)
Asthma History	Yes No		15, 703 (14.1) 95, 325 (85.9)
Allergic History	Yes No		5,177 (4.7) 105,851 (95.3)

Table 2 shows the occupational and organizational factors among study participants. For working hour classification, 15.3% worked less than eight hours, 77.9% worked between eight and 12 h, and 6.8% worked more than 12 h. For the duration of employment, 93.7% of the workers worked less than five years, and the remaining 6.3% worked for more than five years. Among the workers, 22.5% worked in a confined space, and 16.0% worked in a crowded space. The distribution of workers in seven industrial sectors is as follows: manufacturing (38.3%), construction (6.2%), mining and quarry (1.2%), wholesale and retail (28.7%), hotel and restaurant (5.4%), agriculture, fishery, and forestry (4.4%), and utilities (gas, electrical, water and sanitation services) (15.8%). Moreover, 82.9% of workers have access to OSH information and 95.1% have knowledge regarding OSH. The majority (72.5%) had done their training on OSH. Regarding PPE Knowledge, only 63.5% have PPE knowledge, and 67.6% of workers have already undergone training on using PPE. Furthermore, only 56.7% had proper PPE compliance.

**Table 2:** Occupational factors and organizational factors among study participants (*n* =  111,028).

Independent variables		Frequency (%)
Working hours per day	< 8 h 8–12 h > 12 h	17,617 (15.3) 89,497 (77.9) 3,914 (6.8)
Duration of Employment	< 5 years ≥ 5 years	103,978 (93.7) 7,050 (6.3)
Confined space	Yes No	21,949 (19.8) 89,079 (80.2)
Crowded space	Yes No	14,508 (13.0) 96,520 (87.0)
Industrial Sector	Manufacturing	42,528 (38.3)
	Construction	6,924 (6.2)
	Mining and Quarry	1,327 (1.2)
	Wholesale and Retail	31,876 (28.7)
	Hotel and Restaurant	5,941 (5.4)
	Agriculture, Fishery, and Forestry	4,913 (4.4)
	Utilities	17,519 (15.8)
Access to OSH information	Yes No	91,988 (82.9) 19,040 (17.1)
Knowledge on OSH	Yes No	105,589 (95.1) 5,439 (4.9)
Training on OSH	Yes No	80,459 (72.5) 30,569 (27.5)
Knowledge of PPE	Yes No	70,488 (63.5) 40,540 (36.5)
Training on PPE	Yes No	75,102 (67.6) 35,926 (32.4)
Usage of PPE	Yes No	62,926 (56.7) 48,102 (43.3)

**Fig. 1:**
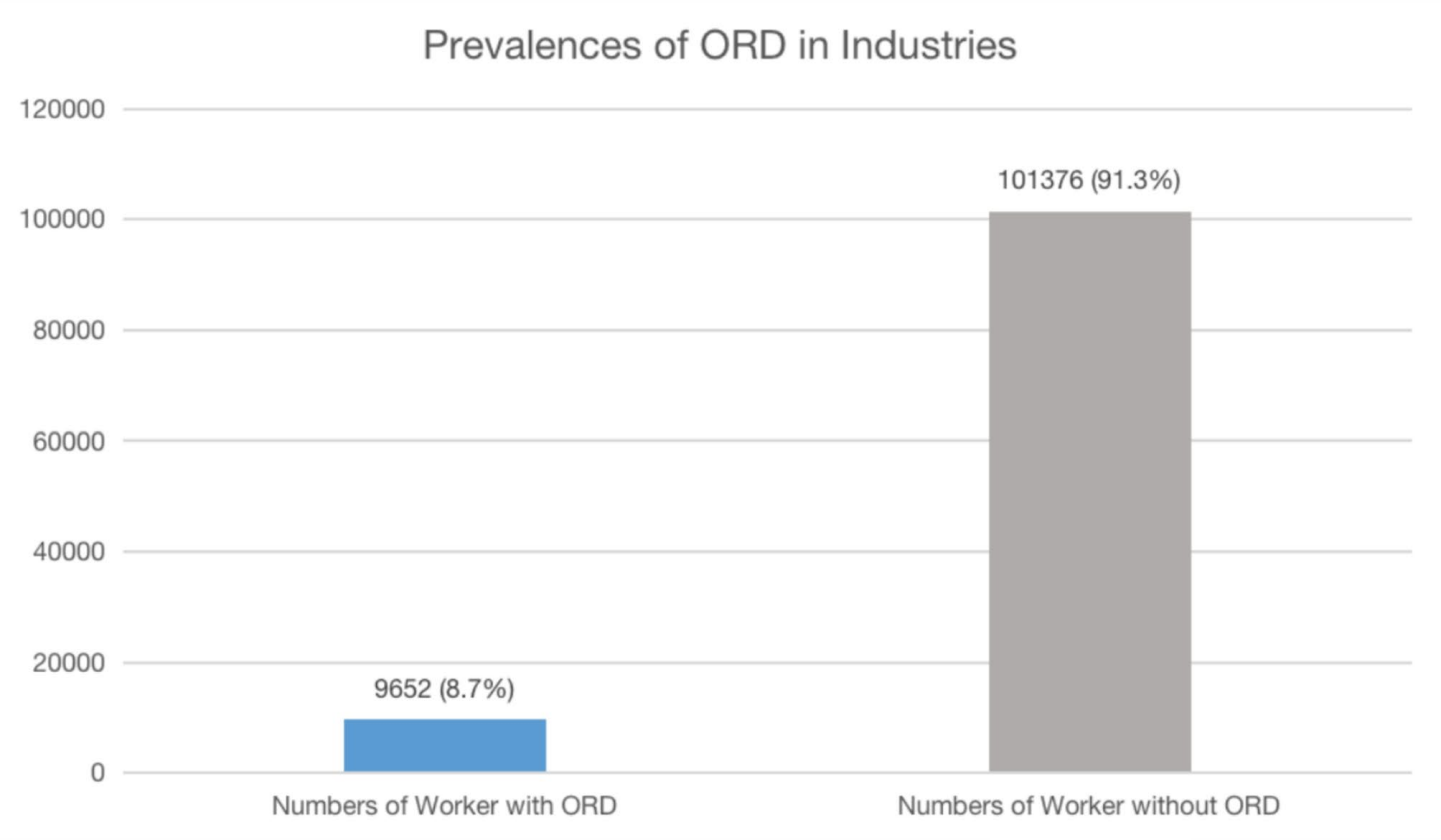
Prevalence of ORD in seven major industries in Malaysia.

Figure 1 shows that the prevalence of ORD among workers from the seven industrial sectors was 8.7%.

**Fig. 2:**
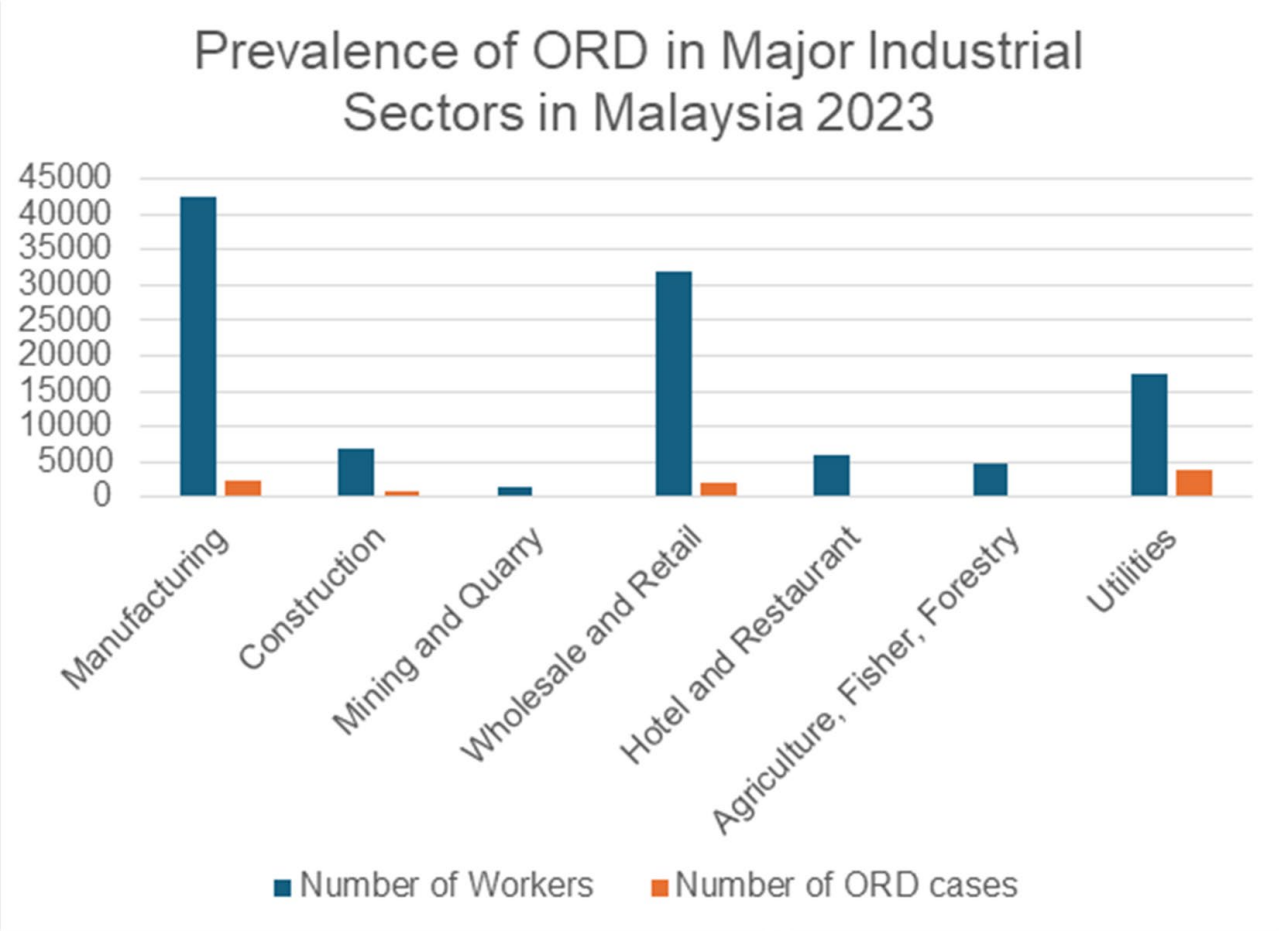
Prevalence of ORD in major industrial sectors in Malaysia.

Figure 2 illustrates the prevalence of ORD in each of the seven sectors in the NODIP database. The highest prevalence was found in manufacturing 2, 478 (5.83%), followed by construction 719 (10.4), mining and quarry 264 (20%), wholesale and retail 1, 986 (6.23%), hotel and restaurant 127 (2.14%), agriculture, fishery, and forestry 150 (3.1%), and utilities 3, 928 (22.4%).

Table 3 presents the statistical results from both simple and adjusted analyses regarding factors associated with (ORD) prevalence among the study participants. From the total of 22 variables analysed, 18 were found to be significant in the univariate analysis. However, after proceeding with the multivariable analysis, only 15 variables retained their significance in the final model where being of older, male, married, of Malaysian nationality, of high education levels of education, smokers, vape users, those with medical illness, those with longer working hours per day, those working in confined or crowded space, those working in industrial sectors; manufacturing, construction, mining and quarry, wholesale and retail, agriculture, fishery and forestry, utilities compared to hotel and restaurant workers, in addition to those lacking of training on PPE and PPE compliance were at higher risk of having ORD. However, being of Malay and Chinese or Indian ethnicity is a protective factor against ORD compared to other ethnic groups. In contrast, three variables did not maintain their significance in the adjusted model which are asthma history, knowledge of OSH, and PPE. All of the variables are justified and discussed in the discussion part.

**Table 3:** Simple and adjusted analysis of the factors associated with ORD prevalence among participants (*n* = 111,028).

Variables		Simple Logistic Regression*		Multiple Logistic Regression**	
		COR (95% CI)	*p*-value	AOR (95% CI)	*p*-value
**Socio-demographic factor**					
Age		1.02 (1.017–1.021)	< 0.001	1.016 (1.013–1.018)	< 0.001
Gender	Male Female	0.69 (0.66-0.69) Ref.	< 0.001 Ref.	1.42 (1.35–1.50) Ref.	< 0.001 Ref.
Marital Status	Married Not Married	1.36 (1.30–1.4) Ref.	< 0.001 Ref.	1.10 (1.046–1.16) Ref.	< 0.001 Ref.
Nationality	Malaysian Non-Malaysian	5.66 (4.94–6.49) Ref.	< 0.001 Ref.	4.48 (3.78–5.32) Ref.	< 0.001 Ref.
Race	Malay Chinese Indian Others	1.77 (1.65–1.89) 1.25 (1.12–1.39) 0.97 (0.79–1.03) Ref.	< 0.001 < 0.001 0.13 Ref.	0.28 (0.25–0.33) 0.30 (0.26–0.32) 0.61 (0.56–0.66) Ref.	< 0.001 < 0.001 < 0.001 Ref.
Level of Education	Tertiary Secondary Primary	2.74 (2.27–3.31) 5.41 (4.49–6.51) Ref.	< 0.001 < 0.001 Ref.	2.57 (2.11–3.14) 1.58 (1.30–1.93) Ref.	< 0.001 < 0.001 Ref.
Smoking Status	Smoker Non-Smoker	1.03 (0.98–1.08) Ref.	0.22 Ref.	1.23 (1.15–1.31) Ref.	< 0.001 Ref.
Vape User	Vape User Non-vape user	1.21 (1.15–1.28) Ref.	< 0.001 Ref.	1.27 (1.19–1.35) Ref.	< 0.001 Ref.
Medical Illness	Yes No	2.11 (2.02–2.20) Ref.	< 0.001 Ref.	1.63 (1.56–1.71) Ref.	< 0.001 Ref.
Asthma History	Yes No	0.9 (0.81–0.99) Ref.	0.046 Ref.	1.01 (0.95–1.07) Ref.	0.74 Ref.
Allergic History	Yes No	1.009 (0.95–1.07) Ref.	0.76 Ref.		
**Occupational factors**					
Working hours/day	> 12 h 8–12 h < 8 h	2.25 (2.01–2.51) 1.42 (1.33–1.51) Ref.	< 0.001 < 0.001 Ref.	2.18 (1.94–2.45) 1.34 (1.26–1.44) Ref.	< 0.001 < 0.001 Ref.
Duration of Employment	*≥* 5 years < 5 years	0.72 (0.65–0.79) Ref.	0.39 Ref.		
Confined Space	Yes No	1.51 (1.44–1.59) < 0.001	< 0.001 Ref.	1.31 (1.24–1.38) Ref.	< 0.001 Ref.
Crowded Space	Yes No	1.56 (1.481.65) Ref.	< 0.001 Ref.	1.58 (1.49–1.67) Ref.	< 0.001 Ref.
Industrial Sectors	Manufacturing	2.83 (2.36–3.39)	< 0.001	3.38 (2.81–4.06)	< 0.001
	Construction	5.30 (4.37–6.42)	< 0.001	10.9 (8.92–13.31)	< 0.001
	Mining and quarry	11.37 (9.11–14.18)	< 0.001	14.81 (11.74–18.68)	< 0.001
	Wholesale and retail	3.04 (2.53–3.64)	< 0.001	3.15 (2.61–3.79)	< 0.001
	Agriculture, fishery, and forestry	1.44 (1.13–1.83)	0.03	3.22 (2.50–4.14)	< 0.001
	Utilities (gas, electrical and water & sanitation services)	13.2 (11.05–15.83)	< 0.001	12.01 (9.98–14.44)	< 0.001
	Hotel and restaurant	Ref.	Ref.	Ref.	Ref
**Organizational factors**					
Access to OSH information	No Yes	1.00 (0.94–1.06) Ref.	0.83 Ref.		
Knowledge of OSH	No Yes	0.79 (0.71-0.88) Ref.	< 0.001 Ref.	0.90 (0.80-1.009) Ref.	0.72 Ref.
Training on OSH	No Yes	0.99 (0.94–1.03) Ref.	0.68 Ref.		
Knowledge of PPE	No Yes	1.3 (1.25–1.36) Ref.	< 0.001 Ref.	0.97 (0.92–1.03) Ref.	0.44 Ref.
Training on PPE	No Yes	1.3 (1.25–1.36) Ref.	< 0.001 Ref.	1.18 (1.12–1.24) Ref.	< 0.01 Ref.
Usage of PPE	No Yes	1.3 (1.25–1.36) Ref.	< 0.001 Ref.	1.07 (1.02–1.12) Ref.	0.02 Ref.

*Simple Logistic Regression significant level set at 0.25.

**Multiple Logistic Regression significant level set at 0.05.

COR: Crude odd ratio.

AOR: Adjusted odd ratio.

Hosmer and Lemeshow test was not significant (p-value > 0.05). This implies that the model is fit. All 22 variables used in the study significantly contribute to the outcome (Omnibus test). The model has a sensitivity of 0.1 and a specificity of 100% and the overall performance of the model is 91%. Pseudo R2, the variable of the model contributes 3.8% (Cox & Snell), and 8.3% (Nagelkerke), to the variability of ORD. The other 96.2% (Cox & Snell), and 91.7% (Nagelkerke) of the variability of ORD is caused by other factors. All variables Variation Inflation Factor (VIF) are less than 10, thus there is no multicollinearity problem in my study. All variables used in the study have no interaction in the analysis of the final model. There is no influential outlier in this study. All the data values were less than 1.

## Discussion

The present study revealed an overall prevalence rate of 8.7% for ORD among study participants with the highest prevalence in the utility sector. The significant factors associated with ORD are age, gender, marital status, nationality, race, levels of education, smoking status, vape user, history of medical illness, working hours per day, working space as confined, crowded space in addition to training on PPE and usage of PPE.

### Prevalence of ORD

Our finding of 8.7% overall ORD prevalence in Malaysian workers positions itself within the global spectrum of occupational respiratory disease burden. This rate is substantially higher than the incidence of 2–5 cases per 100,000 population per year of occupational asthma^15^, but lower than the 51.6% prevalence found among Ethiopian industry workers^16^, industry workers in the UK (22%), Eastern Nepal (21.1%), Hong Kong (27.2%), and Bangladesh (34%)^16^. These disparities likely reflect differences in occupational safety regulations, with Malaysia’s intermediate position mirroring its developing economy status.

### Prevalence of ORD in industrial sectors

The utility sector’s high ORD prevalence (22.4%) aligns with Ahmad & Balkhyour (2020)^17^, who reported 22.7% respiratory symptoms in gas industry workers due to toxic exposures. However, our findings exceed Leon-Kabamba et al. (2020)’s quarry worker study (4.5%)^18^, suggesting regional exposure disparities.

The construction sector emerged as the third highest-risk industry for occupational respiratory diseases (ORD) in this study after utility and mining. This finding aligns with research by da-Silva-Filho et al. (2019)^19^, who reported that 44.4% of construction workers exhibited respiratory symptoms—primarily persistent cough, phlegm production, and wheezing—due to prolonged exposure to airborne hazards. A critical contributor to ORD in this sector is particulate matter (PM) generated from activities like demolition, drilling, and material handling, which accounts for 70–80% of total PM emissions at construction sites^20^. Notably, construction-related PM contains silica, heavy metals, and organic compounds, which are linked to asthma, bronchitis, and COPD^19,20^. These risks underscore the urgent need for stricter dust-control measures and respiratory protection protocols in construction environments.

This study reveals significant sector-specific variations in ORD risk, with manufacturing workers particularly vulnerable to emerging conditions like BADE^21^. Agricultural workers face compounded risks from pesticide/dust exposures, showing 2.3 higher asthma rates^22^. In contrast, wholesale/retail and hospitality sectors demonstrate lower risks^17,23^, aligning with their reduced exposure profiles. These findings underscore the need for sector-specific prevention strategies to address Malaysia’s 8.7% ORD prevalence.

### Socio-demographic factors

#### Age

The workers in the industry have the chance of having ORD rise by 1.6 times with every year of age., this findings is similar to study done among steel workers^7^. Aging is a risk factor and prognostic indicator for acute respiratory distress syndrome, according to Brown, McKelvey^24^, with possible underlying mechanisms contributing to increased risk and severity. As we age, immune-senescence reduces our ability to fight infections, leading to prolonged inflammation that worsens lung injury and accelerates ARDS progression^24^.

#### Gender

The men workers in the industry have the chance of having ORD more than women, this findings is similar to study done in farming cohort^25^. However, women show greater susceptibility and severity in certain respiratory diseases^26^. For example, women are more likely than men to develop chronic obstructive pulmonary disease (COPD)^26^. The disparity is influenced by sociocultural factors, occupational segregation, and underestimation of women’s occupational exposures and related diseases^25,26^.

#### Married status

Research indicates that married individuals have a higher risk of getting ORD than unmarried. Saupin, Hayati^27^ found that Malaysian construction workers who were exposed to dust, and experienced ORD symptoms along with divorce, separation, or being widowed are more likely to have ORD than married or single. The study suggests further research is needed to better understand the link between respiratory health and marital status among industrial workers^27^.

#### Nationality

Malaysian respondents are more susceptible to experiencing ORD compared to non-Malaysians. The high prevalence of Malaysian respondents (89%) compared to foreigners (11%) further reinforces this finding. Study conducted by^11^ highlighted that workers in Malaysia face significant occupational health risks, especially in regions undergoing fast industrialization. The study emphasised that respiratory infections are a significant health concern among Malaysian workers, underscoring the need for improved workplace safety measures and healthcare interventions^11^.

#### Races

The analysis suggests that Chinese, Indian, and Malay workers appear to be less likely to develop occupational lung diseases (odd ratios 0.28, 0.3, and 0.61, respectively). Most studies on racial disparities in occupational lung diseases focus on groups such as African-Americans, Hispanics, and Whites, showing that minority groups in the US often have higher occupational exposures and worse respiratory outcomes due to socioeconomic and environmental factors rather than inherent racial susceptibility^28,29^. Research indicates that racial/ethnic disparities in occupational exposure and respiratory disease risk are largely driven by social determinants such as job types, income, education, and access to healthcare, rather than race itself as a biological risk factor^30^.

#### Levels of education

There was a significant relationship between the level of education and ORD, whereby people with a higher education level were more likely to develop ORD. This finding is contrary to the expectations of the theory that increased education reduces the occurrence of ORD^31,32^. However, a study among schoolteachers found that teachers are at high risk of ORD^33^. One possible explanation for the connection is that employees with tertiary education might be engaged in hazardous occupations, increasing their chances of being in contact with toxic substances. These exposures considerably raise their vulnerability to respiratory diseases; hence the call for tight safety measures and constant health check-ups.

#### Smoking status

From the study, the odds of a respondent being a smoker are higher compared to those who do not smoke. Smoking impairs immune function, reduces mucociliary clearance, and causes airway inflammation, which heightens the risk of acute respiratory infections and worsens outcomes in occupationally exposed workers^34^. This finding is consistent with multiple research that highlights the substantial influence of smoking on the respiratory well-being of industrial workers^7,35^. This highlights the importance of enacting smoking cessation programmes and establishing safety measures in the workplace^27^.

#### Vaping status

Vaping significantly increases the likelihood of developing ORD, consistent with research showing its adverse respiratory effects. A study conducted a comprehensive investigation into cases of lung injury associated with the usage of e-cigarettes or vaping products, commonly referred to as EVALI^36^ The study highlighted that a significant fraction of EVALI patients disclosed the consumption of items containing tetrahydrocannabinol (THC), leading to a major proportion of them requiring hospitalisation as a result of ARDS^36^.

#### Medical illness

Regarding studies on occupational respiratory diseases, research by De Matteis, Heederik^15^ suggests that individuals with previous medical history are significantly more susceptible to acquiring ORD. This observation aligns with numerous other studies that have emphasised the correlation between existing medical conditions and respiratory well-being in work settings. Additionally, it emphasised the hazards linked to work risks for employees who had preexisting respiratory conditions^15,37,38^.

### Occupational factors

#### Working hours per day

Employees working more than 12 h a day stand the highest risk of developing the disease, followed by those working less hours. Long working hours were a risk factor that was brought out clearly by the COVID-19 pandemic by Mhango, Dzobo^39^ who discovered that long working hours predisposed healthcare workers to COVID-19. This means that several respiratory diseases are likely to occur as a result of high and prolong exposure to inhalable pollutants arising from long working hours^39^.

#### Workspace (confined, crowded)

Confined and crowded environments at the workplace increase the spread of respiratory diseases among employees. Moon and Ryu^40^ conducted a meta-analysis and found that workplaces are significant areas for respiratory infection transmission. The study concluded that it might increase the risk of ORD because of poor ventilation, close contact between workers, and shared indoor air. These are the basic characteristics of enclosed workplaces that increase the risk of transmission of respiratory diseases^40,41^.

### Organizational factors

#### Training in PPE

Numerous study shows that workers who underwent PPE training have a lower risk of developing ORD. Studies conducted between 2020 and 2024 during emergence of pandemic COVID19 have stressed the significance of PPE training in enhancing the safety levels of employees and preventing the spread of respiratory diseases at the workplace^42^.

#### Usage of PPE

Ensuring compliance with PPE protocols is essential for protecting worker’s health and reducing ORD. Houghton, Meskell^43^ studied factors affecting industrial workers’ compliance with PPE and highlighted the importance of using PPE and mandatory training to enhance compliance. The challenges identified insufficient training and inconsistent utilisation of PPE, leading to an elevated infection rate among staff. Hence, there is an urgent need for adequate training and instruction to encourage the appropriate use of PPE^43^.

### Study limitation

While this study provides valuable insights, it is crucial to acknowledge its limitations. The utilisation of self-reported data obtained through an online questionnaire increases the probability of recall bias and social desirability bias. Furthermore, it is important to acknowledge that the use of a cross-sectional design in this study restricts the capacity to establish causal connections between suspected ORD and its associated components. Additionally, as this is a test specifically for symptoms, it is recommended to undergo further screening if there is suspicion of an underlying medical condition. Employing spirometers and lung function testing is advised as supplementary means to assess the respiratory well-being of the workforce. Longitudinal or prospective designs are more appropriate for future research on the temporal relationship between occupational exposures and the development of suspected ORD^44^.

### Recommendation

To address the high prevalence of occupational respiratory diseases (ORD), prioritize further investigation, risk assessment and interventions in high-risk sectors such as manufacturing, utilities, construction, and mining by enforcing strict occupational health and safety (OSH) regulations, improving workplace ventilation, and mandating proper use of personal protective equipment (PPE). Conduct regular training on PPE use, monitor compliance, and offer incentives for adherence. Implement health-focused measures, including smoking and vaping cessation programs and regular medical screenings for at-risk workers, particularly those with pre-existing illnesses. Redesign workspaces to reduce crowding, advocate for policies limiting long working hours, and tailor interventions for older, male, and married workers who are at higher risk. Increase awareness of OSH through targeted campaigns and develop sector-specific guidelines while ensuring inclusivity across ethnic groups. Establish feedback mechanisms for reporting unsafe conditions and continuously update the NODIP database to monitor ORD trends and intervention effectiveness.

## Conclusion

Based on the results, it can be concluded that occupational respiratory diseases (ORD) are highly prevalent in sectors such as manufacturing, utilities, construction, and mining, highlighting the critical need for targeted interventions. Key risk factors include demographic characteristics (older age, male, and married status), lifestyle habits (smoking and vaping), occupational conditions (long working hours, confined or crowded spaces), and inadequate training and compliance with personal protective equipment (PPE) usage. Conversely, being of Malay, Chinese, or Indian ethnicity appears to be a protective factor against ORD. These findings underscore the importance of sector-specific preventive measures, enhanced workplace safety protocols, and comprehensive health promotion programs to mitigate risks and reduce the overall burden of ORD.

## Electronic supplementary material

Below is the link to the electronic supplementary material.

Supplementary Material 1

## Data Availability

The datasets used and/or analysed during the current study are available from the corresponding author upon reasonable request.
