# Supplementary material for: Prevalence of occupational respiratory disease and its determinants among workers in major industrial sectors in Malaysia in 2023
Source: Sci Rep. 2025 Jul 16;15:25807. doi: 10.1038/s41598-025-10365-8 (PMC12267597; doi:10.1038/s41598-025-10365-8)
Supplement: Supplementary file 1 — Supplementary Material 1 [file 41598_2025_10365_MOESM1_ESM.docx]

**Occupational Respiratory symptoms**

This question is not based on Fever, cough or Cold

**Question 1.0**

**1.0 Cough**

1.1 Do you usually cough at all on getting up, or first thing in the morning?

1. ☐ Yes 2. ☐ No

1.2 Do you usually cough at all during the rest of the day or at night?

1. ☐ Yes 2. ☐ No

If **NO** to question 1.1 and 1.2 , proceed to question 2.0

1.3 Do you have a persistent cough most days, for 3 months during a year?

1. ☐ Yes 2. ☐ No

1.4 Do you only cough at work time and it goes away when you get home?

1. ☐ Yes 2. ☐ No

**Question 2.0**

**2.0 Phlegm**

2.1. Do you usually bring up phlegm or first thing in the morning?

1. ☐ Yes 2. ☐ No

2.2 Do you usually bring up phlegm at all during the rest of the day or at night?

1. ☐ Yes 2. ☐ No

If **NO** to the question 2.1 and 2.2, proceed to question 2.3

2.3. Do you usually bring up phlegm like this on most days for 3 consecutive months or more during the year?

1. ☐ Yes 2. ☐ No

2.4 Have you had episodes of (increased*) cough and phlegm lasting for 3 weeks or more each year?

1. ☐ Yes 2. ☐ No

**Question 3.0.**

**3.0 Chest Tightness**

3.1 Have you ever felt chest tightness or difficulty in breathing ?

1. ☐ Yes 2. ☐ No

If **NO**, proceed to question 4

3.2 On the first day back to work

1. ☐ Yes 2. ☐ No

3.3 On another day

1. ☐ Yes 2. ☐ No

**Question 4.0**

**Breathlessness**

4.1Are you troubled by shortness of breath when hurrying on the level or walking up a slight hill?

1. ☐ Yes 2. ☐ No

4.2 Do you have to walk slower than people of your age on level because of breathlessness?

1. ☐ Yes 2. ☐ No

4.3 Do you ever have to stop for breath when walking at your own pace on the level?

1. ☐ Yes 2. ☐ No

4.4 If **YES,** does shortness of breath only occur on the first day of return to work?

1. ☐ Yes 2. ☐ No

British Medical Research Council (BMRC)

(Cotes et al., 1988)

| Occupational Respiratory Disease | **The presence of one or more symptoms and at least one work-related domain in the survey confirms the presence of ORD.** |
| --- | --- |
|  | At least 1 of these are recognized to has **Occupational Respiratory Disease:** |

1. The symptoms were caused by work hazards
2. The symptoms worsen at work
3. the symptoms improve during off days
4. coworkers demonstrate the same symptoms

***LaDou & Harrison et al, (2014), Current Diagnosis & Treatment: Occupational & Environmental Medicine***
